# Efficient production of fully active, SARS-CoV-2-priming, wildtype TMPRSS2 ectodomain via co-expression of HAI-2 allows for both auto- and cross-activation mechanisms

**DOI:** 10.1042/BCJ20253453

**Published:** 2025-12-17

**Authors:** Barbara Végh, Andrea Kocsis, József Dobó, Júlia Balczer, Bence Kiss, Gábor Pál, Péter Gál

**Affiliations:** 1 EvolVeritas Biotechnology Ltd., Budapest, Hungary; 2 Institute of Molecular Life Sciences, HUN-REN, Research Centre for Natural Sciences, Budapest, Hungary; 3 Department of Biochemistry, Eötvös Loránd University, Budapest, Hungary

**Keywords:** HAI-2, kunitz-type inhibitor, SARS-CoV-2, TMPRSS2, zymogen auto-activation

## Abstract

Transmembrane protease serine (TMPRSS)2 is a cell surface host protease, which plays a decisive role in viral infections. This trypsin-like enzyme cleaves the spike protein of coronaviruses [e.g. severe acute respiratory syndrome coronavirus 2 (SARS-CoV-2)] and the hemagglutinin protein of influenza viruses, enabling viral membrane fusion and subsequent viral cell entry. Consequently, TMPRSS2 is an attractive therapeutic target, and it is of utmost importance to produce wildtype, enzymatically active recombinant TMPRSS2 for drug development purposes. We present the first successful strategy for the expression of wildtype and proteolytically fully active ectodomain of human and Syrian hamster TMPRSS2 in mammalian cells. To achieve that, we co-expressed the TMPRSS2 ectodomain with its natural inhibitor, hepatocyte growth factor activator inhibitor-2, which yielded substantial amounts of secreted, native protease. Most of the recombinant TMPRSS2 was secreted as zymogen, which was activated during purification. The purified activated recombinant TMPRSS2 ectodomain cleaved synthetic and protein substrates with high efficiency (*k*cat/*K_M_* in the 10^4^–10^6^ M^-1^s^-1^ range). To study the mechanism of auto-activation, we expressed zymogen TMPRSS2 mutants as well. We found that the zymogen is an ideal substrate for the active protease as it cleaves it extremely efficiently. We also showed that zymogen TMPRSS2 itself has a weak proteolytic activity, which can initiate the auto-activation process. We demonstrated that a related cell surface protease, TMPRSS13, is also able to activate zymogen TMPRSS2. Our findings prove both a zymogen trans(auto)-activation as well as a TMPRSS13-based cross-activation mechanism, the latter supporting that type II transmembrane serine proteases can form a pericellular proteolytic cascade.

## Introduction

Serine proteases (SPs) constitute one of the largest families of proteolytic enzymes and play pivotal roles in diverse physiological and pathological processes such as embryonic development, immune defense, and tumorigenesis [1]. Transmembrane SP TMPRSS2 (transmembrane protease serine 2) was identified and cloned in 1997 [2], and it has recently gained enormous interest due to its cardinal role in the severe acute respiratory syndrome coronavirus (SARS-CoV)-2 infection [3]. Although the normal physiological function of mammalian TMPRSS2 is still not fully known, it participates in various pathological processes. During virus infection, many coronaviruses, including SARS-CoV-1, SARS-CoV-2, Middle East respiratory syndrome coronavirus (MERS-CoV), and human coronavirus 229E (HCoV-229E), as well as influenza viruses, use the host protease TMPRSS2 as a tool to enter the human cells. TMPRSS2 cleaves coronavirus spike (S) protein and influenza virus hemagglutinin, thereby allowing the fusion between viral envelope and host cell membranes and promoting virus genome delivery. Inhibition of the proteolytic activity of TMPRSS2 is an attractive therapeutic option for preventing and treating coronavirus and influenza virus infections [4,5]. TMPRSS2 expression is up-regulated in response to androgens in prostate cancer. It was suggested that TMPRSS2, as a member of a pericellular proteolytic cascade, induces cancer cell invasion, tumor growth, and metastasis [6,7]. Its increased expression in prostate cancer indicates that TMPRSS2 might be a therapeutic target for prostate cancer [8]. An entirely different involvement of the *TMPRSS2* gene has been detected in a high-frequency chromosomal aberration in prostate cancer, where fusion of the promoter region of the *TMPRSS2* gene with the erythroblast-specific-related gene (*ERG*) results in androgen-driven overexpression of this oncogenic transcription factor [9].

As mentioned above, the normal physiological function of TMPRSS2 is still elusive. While in the case of most of the similar type II transmembrane SPs (TTSPs), their genetic knockout is lethal or results in the development of severe disease conditions, TMPRSS2 KO mice [10] and pigs [11] are viable, fertile, and do not show any abnormal phenotype. Moreover, the KO animals showed a reduced inflammatory response against influenza A virus infection [12,13]. Nevertheless, evolutionary conservation of TMPRSS2 suggests that it should have important, albeit not yet defined, biologic functions.

The host protease TMPRSS2 is a member of the TTSP family. The TTSPs form the largest group of membrane-anchored SPs, comprising 17 members in humans [14]. The members of the TTSP family share common protein structures, including an N-terminal cytoplasmic domain, a single-path transmembrane (TM) domain, an extracellular linker region with a variety of protein–protein interaction domains, and at the C terminus, a SP domain with chymotrypsin (S1) fold. The TTSPs have trypsin-like substrate specificity, hydrolyzing the peptide bonds after basic amino acid residues (Lys, Arg). They are synthesized as one-chain zymogens and become activated via limited proteolysis within the SP domain. Several lines of evidence suggest that TTSPs form a proteolytic cascade on the cell surface, like the blood coagulation and the complement system in the blood [6,15]. It was shown that TMPRSS2 activates matriptase, a TTSP whose uncontrolled activation contributes to cancer development. It was suggested earlier that TMPRSS2 can auto-activate, but the exact mechanism has not been revealed [16,17].

Since TMPRSS2 has great therapeutic relevance, it is of utmost importance to produce it recombinantly in a wildtype and fully active form to facilitate drug development. According to the literature, and based on our own experience, recombinant expression of TMPRSS2 is extremely challenging.

As Syrian hamsters have been used as model animals for infection with respiratory viruses, including SARS-CoV-2 [18], we decided to express the extracellular fragment (ectodomain) of both human and Syrian hamster (*Mesocricetus auratus*) TMPRSS2 in mammalian cells. We managed to produce recombinant TMPRSS2 ectodomain with good yield by co-expressing it with its cognate inhibitor, hepatocyte growth factor activator inhibitor (HAI)-2. The purified TMPRSS2 ectodomain showed high enzymatic activity.

We also aimed to clarify the exact mechanism of zymogen activation of these proteases; therefore, in addition to the wildtype form, we also expressed two kinds of zymogen mutants. We showed that TMPRSS2 has a high autoactivating capacity. The wildtype active protease activates the zymogen enzyme extremely efficiently. We also demonstrated that the zymogen has a low but detectable proteolytic activity, which is sufficient to start the autoactivation process. Moreover, we also showed that a related host TTSP, TMPRSS13, is also able to activate the zymogen TMPRSS2.

## Results

### Recombinant expression of the ectodomain of TMPRSS2

**Figure 1:**
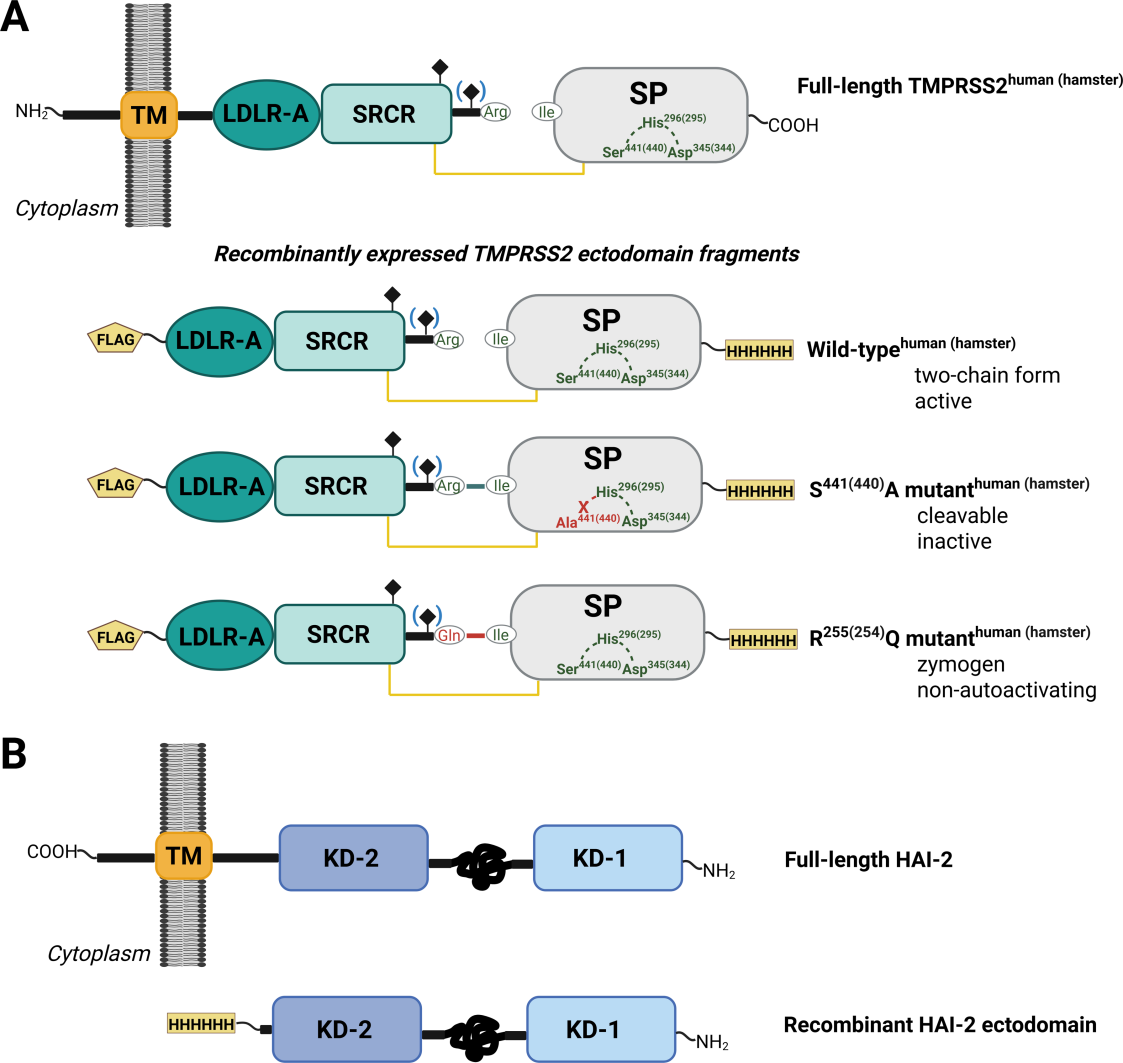
Schematic representation of the domain structures of TMPRSS2 and HAI-2. (**A**) Wildtype and mutant forms of human and hamster TMPRSS2 ectodomain were expressed in HEK293F cells. The recombinant TMPRSS2 fragments include an N-terminal FLAG tag, a low-density lipoprotein receptor type A domain (LDLR-A), a class A scavenger receptor cysteine-rich domain (SRCR), a trypsin-like serine protease domain (SP), and a C-terminal histidine tag. The catalytic triad residues—serine, histidine, and aspartate—are shown in green, with human numbering followed by hamster numbering in parentheses. Mutated residues are highlighted in red, while the glycosylation site, which is absent in hamster TMPRSS2, is indicated in blue parentheses. (**B**) The HAI-2 inhibitor was expressed both as a full-length wildtype transmembrane protein and as a recombinant extracellular fragment in HEK293 cells. HAI-2 consists of a C-terminal type I transmembrane domain (TM) and two Kunitz domains (KD-1 & -2) connected by a disordered region. The recombinant ectodomain carries a C-terminal histidine tag. TM, transmembrane domain; KD, Kunitz domain.

The extracellular region (ectodomain) of human TMPRSS2 consists of 3 domains: a low-density lipoprotein receptor domain class A (LDLR-A) domain (residues 112–149), a scavenger receptor cysteine-rich domain (SRCR) domain (residues 150–242), and an SP domain (residues 243–492) (Figure 1A). As we have decades of experience in refolding multidomain human blood-borne SPs [e.g. C1r, C1s, mannose-binding lectin-associated serine protease (MASP)-1,-2,-3] produced in bacterial expression systems [19–22], first we tried to produce human TMPRSS2 in an *Escherichia coli* expression system. The bacteria produced the recombinant proteins in inclusion bodies; however, we could not renature them after solubilization. We tried to renature different domain constructs (e.g. the whole ectodomain, SRCR-SP, the SP domain alone), with or without a tag (e.g. N-terminal MBP tag), using various renaturation protocols, but we failed to get a detectable amount of enzymatically active TMPRSS2. Human TMPRSS2 contains an unpaired cysteine (Cys379) residue, which is otherwise engaged in a disulfide bond in the related TTSPs. In the human TMPRSS2, however, a threonine (Thr447) occupies the place of the other cysteine residue of the corresponding disulfide bridge. We mutated this threonine to cysteine in the DNA construct to facilitate renaturation. However, the restoration of the disulfide bridge did not improve the efficiency of the renaturation process (data not shown).

Next, we tried to express the ectodomain construct in mammalian cells. We subcloned the ectodomain constructs into the pSecTag2c expression vector [23]. The resulting recombinant constructs contained the CMV enhancer and promoter, the IgG kappa chain leader sequence, the FLAG tag before the TMPRSS2 sequence, and the Myc- and His-tags at the C-terminus of the protein. As we also wanted to study the mechanism of the zymogen (auto)activation of TMPRSS2, we made two types of zymogen mutant constructs.

TMPRSS2 has a typical trypsin-like SP activation site, Arg255-Ile256, where the peptide bond is hydrolyzed by limited proteolysis during activation, liberating the SP domain having Ile256 at its N-terminus. After cleavage, a disulfide bond holds together the activated SP domain and the N-terminal non-catalytic domains.

In one of the zymogen mutant constructs (RQ mutant), we mutated the Arg255 to Gln (Arg254Gln in the case of hamster TMPRSS2), rendering this site resistant to trypsin-like SPs. While in this regard, the mutated site is stable, it can be processed by the metalloprotease, thermolysin. We used these stable zymogen constructs as enzymes to test the catalytic properties of the otherwise unstable wildtype zymogens.

In the other zymogen mutant construct (SA mutant), we mutated the active site serine to alanine. The Ser441Ala construct (Ser440Ala in the case of hamster TMPRSS2) is enzymatically inactive, but it can be cleaved at the Arg255–Ile256 bond by a trypsin-like SP. These constructs are therefore used as substrates in our experiments.

**Figure 2:**
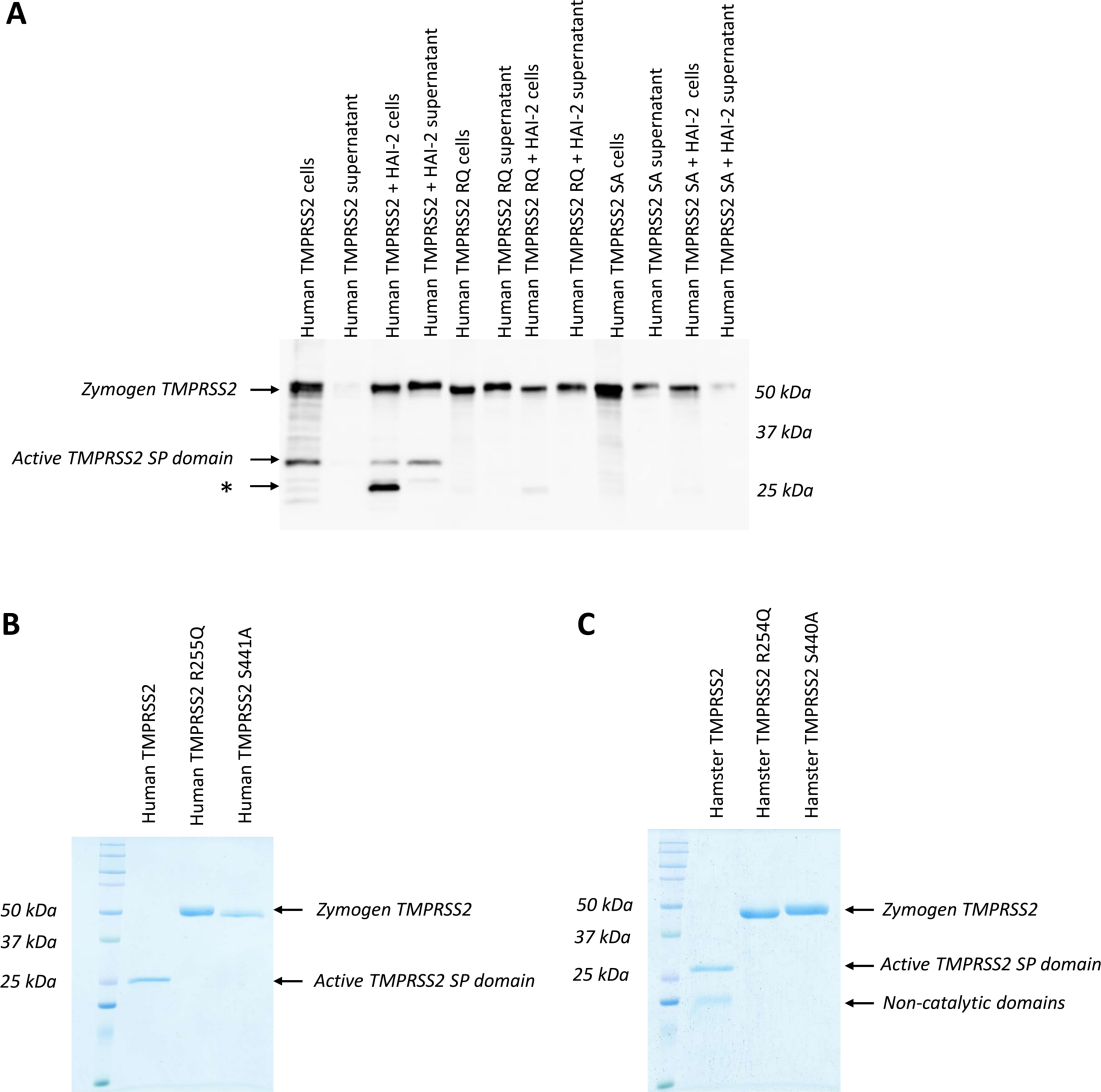
Recombinant expression of wildtype and zymogen mutant TMPRSS2 ectodomains in HEK293F cells. (**A**) Western blot analysis of the expression of human wildtype TMPRSS2, TMPRSS2 R255Q, and TMPRSS2 S441A mutants in the presence or absence of the HAI-2 inhibitor in the cell culture supernatant and in the lysates of FreeStyle HEK293F cells. The protein expression was carried out in a volume of 30 ml. Production was terminated after four days at a cell concentration of approximately 3 × 10^6 cells/ml. The cell suspension was centrifuged, and the cell pellet was resuspended in an equal volume of PBS buffer. During sample preparation, a 4× concentrated reducing sample buffer was applied. Fifteen microliters of each sample were loaded into each well of the gel. Detection was performed using an anti-human TMPRSS2 monoclonal antibody, followed by chemiluminescent detection. The asterisk (*) indicates a proteolytic fragment of TMPRSS2 that was protected from the complete degradation by HAI-2. (**B**) Coomassie-stained reducing SDS-PAGE of purified recombinant human and (**C**) Syrian hamster wildtype, R255/254Q, and S441/440A mutant TMPRSS2 ectodomains. The proteins were expressed in the FreeStyle HEK293F cells, isolated from the cell culture supernatant, purified to homogeneity by immobilized metal affinity, and size-exclusion chromatography and concentrated to 0.2–1 µg/µl.

For recombinant protein production, FreeStyle 293 F (HEK293F) cells were transiently transfected with the DNA constructs. Cell cultures were harvested at 96 hours (four days) post-transfection, and the recombinant protein content was analyzed. To our surprise, in the case of the wildtype construct, we could detect only traces of recombinant TMPRSS2 in the cell culture supernatant (Figure 2A). Most of the TMPRSS2 protein and its degradation products remained inside the HEK293F cells. In contrast to that, the two zymogen mutants could be detected and isolated from the supernatant in a zymogen state with a reasonable yield (R255Q: 1.2 mg/l, S441A: 0.2 mg/l) (Figure 2A and B). Comparison of these results suggested that the wildtype zymogen TMPRSS2 construct became auto-activated and eventually degraded inside the cells.

The physiological inhibitors of the TTSPs are the HAI-1 and -2 [24]. These membrane-bound inhibitors contain a pair of Kunitz-type inhibitor domains and tightly regulate the activity of the cell surface trypsin-like SPs. In the case of matriptase, it was shown that HAI-2 stabilizes the protease along the secretory pathway, possibly via preventing zymogen activation and saving the cells from the harmful uncontrolled proteolytic activity of the activated enzyme [25]. In order to prevent TMPRSS2 auto-activation inside the cells, we decided to co-express TMPRSS2 with HAI-2 in the HEK293F cells. For the HAI-2 co-expression, we used the full-length HAI-2 gene (Figure 1.B) inserted into the pcDNA3.1 expression vector. We co-transfected the HEK293F cells with the pSecTag2c-TMPRSS2 ectodomain and pcDNA3.1-HAI-2 DNA constructs. Since the TMPRSS2 ectodomain does not contain the TM domain, it is secreted into the medium. In contrast, the full-length HAI-2 protein remains attached to the cell membrane and can therefore be easily separated from the secreted TMPRSS2 ectodomain.

This novel strategy finally worked and yielded a significant amount of wildtype recombinant TMPRSS2 ectodomain (1.2 mg/l) that could be isolated from the cell culture supernatant (Figure 2.A,B). The cells bearing recombinant HAI-2 were removed by centrifugation, and the secreted recombinant TMPRSS2 ectodomain was purified from the supernatant by using chromatographic methods [gel filtration and immobilized metal affinity chromatography (IMAC)] (Figure 2.B). Most of the recombinant TMPRSS2 ectodomain was secreted as a one-chain zymogen molecule and became fully activated during purification and concentration.

The Syrian hamster TMPRSS2 ectodomain constructs were produced by a similar way in HEK293F cells (wildtype: 2.0 mg/l, R254Q: 3.2 mg/l, S440A: 4.0 mg/l) (Figure 2.C). In the case of the hamster TMPRSS2, we also used CHO cells for recombinant protein expression and got a reasonably high expression yield (data not shown). We also prepared the ectodomain of a related TTSP, human TMPRSS13, using a similar method with a yield of 2.3 mg/l.

### Characterization of the enzymatic properties of the recombinant TMPRSS2

We characterized the enzymatic activity of the recombinant proteases both on synthetic and protein substrates. We determined the kinetic parameters of the enzymatic reactions (*k*_cat_ and *K*_*M*_) using the synthetic substrate Boc-Gln-Ala-Arg-AMC for both the human and the hamster wildtype TMPRSS2 constructs (Table 1). These constructs hydrolyzed the synthetic substrate with high efficiency. In contrast to that, the zymogen mutants (R255Q and S441A) did not show any detectable activity on the synthetic substrate.

**Table 1:** Kinetic parameters of wildtype human and hamster TMPRSS2 ectodomains.

	Human TMPRSS2	Hamster TMPRSS2
(a) *k*_cat_ (s^-1^)	(6.14 ± 0.55) × 10^1^	(7.68 ± 0.69) × 10^2^
*K*_*M*_ (M)	(6.71 ± 0.55) × 10^-5^	(1.21 ± 0.12) × 10^-4^
*k*_cat_/*K*_*M*_ (M^-1^ s^-1^)	(9.15 ± 0.08) × 10^5^	(6.38 ± 0.52) × 10^6^
(b) *K*_*I*_ (HAI-2; pM)	4.6 ± 0.5	0.7 ± 0.1

(**a**) *K*_*M*_ and *k*_cat_ values were measured using the fluorogenic substrate Boc-Gln-Ala-Arg-AMC and represent the mean of three independent measurements. (**b**) Inhibitory constants (*K*_*I*_) for HAI-2 with the wildtype human and Syrian hamster TMPRSS2 were determined at an initial enzyme concentration of 50 pM. Kinetic assays were performed independently in triplicate, and standard deviations for each dataset are indicated

The cognate inhibitor HAI-2 efficiently and completely abolished the enzymatic activity of the recombinant wildtype TMPRSS2 ectodomain constructs (Table 1, Supplementary Figure S1).

**Figure 3:**
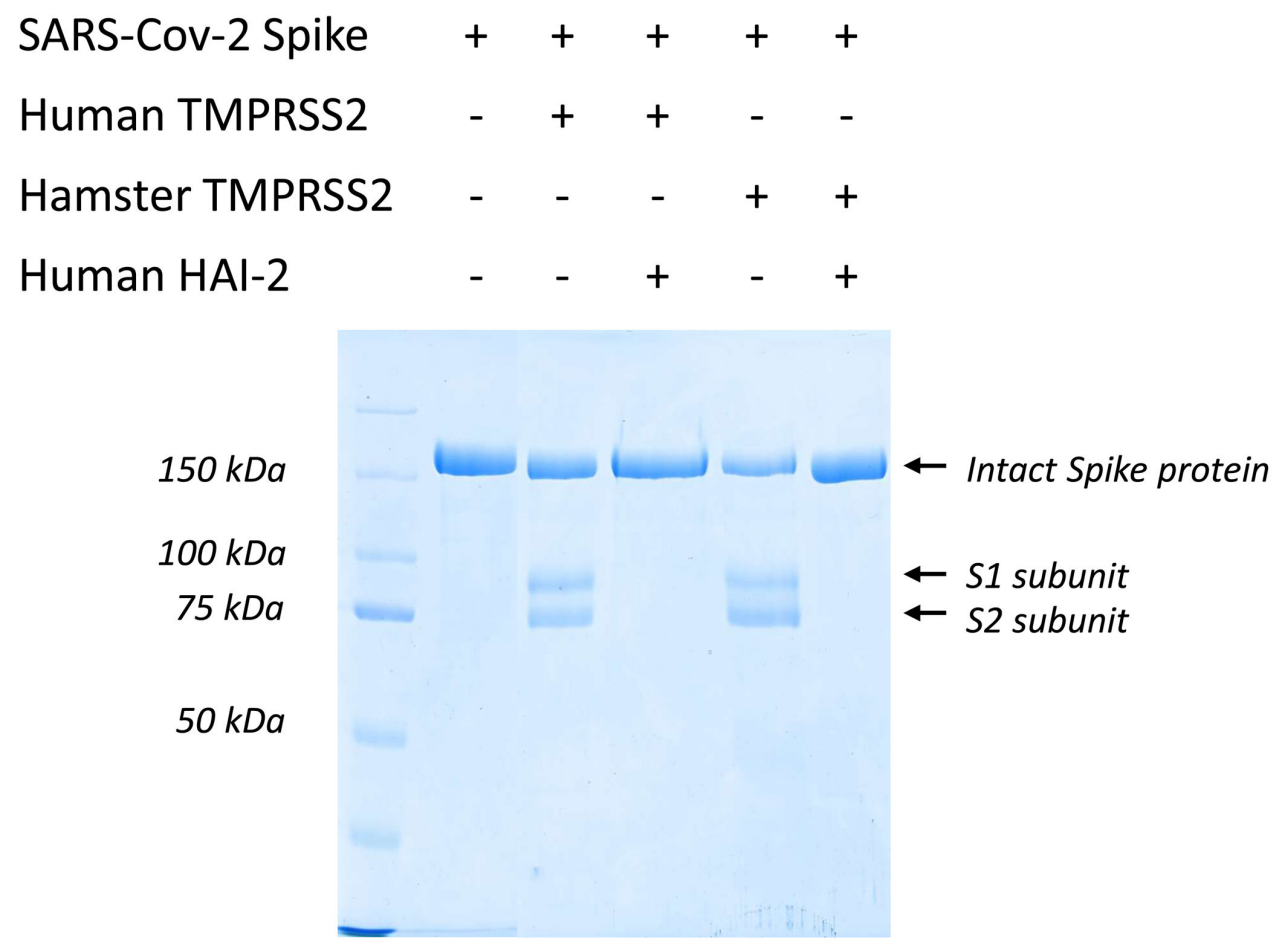
Recombinant TMPRSS2 efficiently cleaves SARS-CoV-2 spike protein at the S1/S2 site. The proteolytic activity of the human and Syrian hamster recombinant TMPRSS2 ectodomains was tested on SARS-CoV-2 spike (**S**) protein substrate and monitored by Coomassie-stained reducing SDS-PAGE. The reaction mixture contained 10 µM trimeric S protein and 33 nM human or 100 nM hamster TMPRSS2, and was incubated for 1 hour at 37°C. Both the human and the Syrian hamster recombinant TMPRSS2 specifically cleaved the SARS-CoV-2 S protein at the S1/S2 site. The proteolytic activity was completely abolished in the presence of 1 µM HAI-2. *Lane1,* Kaleidoscope (250, 150, 100, 75, 50, 37, 25, 20, 15, 10 kDa).

We also checked the proteolytic activity of the wildtype enzymes on the important protein substrate: SARS-CoV-2 S protein. We produced the recombinant ectodomain of the SARS-CoV-2 S protein in insect cells in the trimeric form [26]. Recombinant human and hamster TMPRSS2 ectodomain (33 nM and 100 nM, respectively) cleaved intact S protein ectodomain (10 µM) at the S1/S2 site, generating the two fragments: S1, which runs at about 90 kDa apparent molecular weight in the SDS-PAGE, and S2, which runs at about 75 kDa (Figure 3). After 1 hour of incubation at 37°C, we detected the discrete bands corresponding to the S1 and S2 fragments of the viral S protein. The cleavage at the S1/S2 site was very effective and highly specific, indicating that the S1/S2 site is by far the most preferred cleavage site for the TMPRSS2 protease in the S protein. Extended incubation under these conditions leads to the gradual degradation of both the S1 and the S2 fragments. Our results are in agreement with those of Fraser et al. [27] who also demonstrated that the multibasic S1/S2 cleavage site is by far the most recognized one by TMPRSS2. These results support the notion that instead of furin dependence, the airway-expressed TTSPs are mainly responsible for recognition and cleavage of SARS-CoV-2 S protein facilitating viral infection [28,29].

### Zymogen activation

One of our main goals was to clarify the auto-activation mechanism of zymogen TMPRSS2. Zymogen auto-activation of most trypsin-like SPs takes place in two steps [30]. In the first step, when only one-chain zymogen molecules are present, there is a slow lag phase during which the first active two-chain molecules are formed. The activated two-chain molecules then, in the second step, cleave and activate more zymogens quickly and efficiently.

In the case of trypsin-like SPs, the first step can follow either a zymogen trans(auto)-activation mechanism, when one-chain zymogen form, through its low proteolytic activity, cleaves another one-chain zymogen of the same type generating the first two-chain molecules [31,32], or it can follow a cross-activation mechanism executed by a different protease (e.g. when enterokinase cleaves trypsinogen in the small intestine) [33].

**Figure 4:**
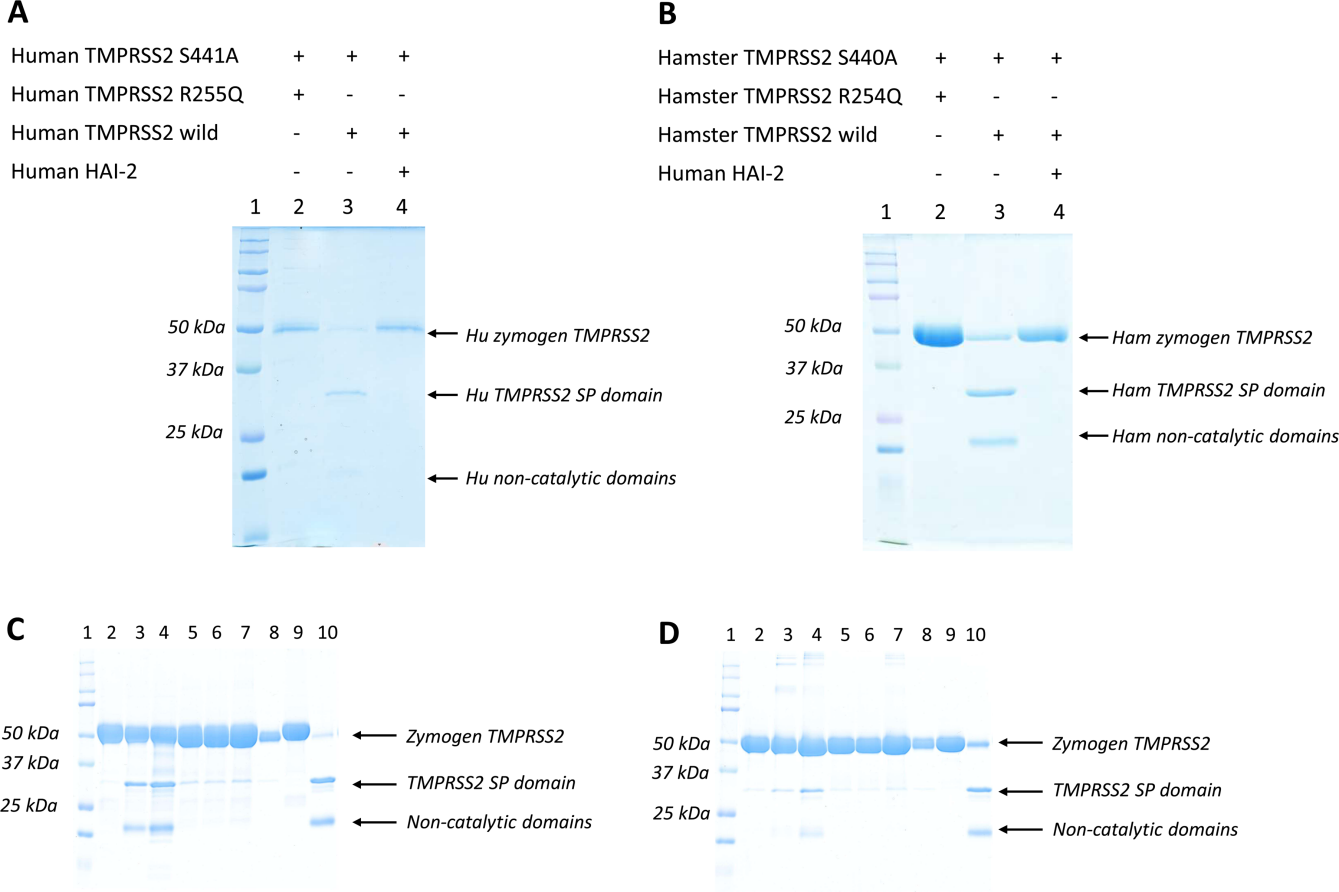
Autoactivation of human (Hu) and Syrian hamster (Ham) TMPRSS2. (**A**) The proteolytic activity of the wildtype and zymogen R255Q mutant human TMPRSS2 was checked on the inactive one-chain TMPRSS2 S441A protein substrate. The reaction mixtures were incubated for 1 hour at 37°C. The wildtype enzyme cleaved the S441A mutant protein at a 1:1 molar ratio, and this cleavage was completely inhibited by HAI-2. In contrast to that, the R255Q mutant did not show any proteolytic activity under these conditions. *Lane 1*, Kaleidoscope markers (250, 150, 100, 75, 50, 37, 25, 20, 15, 10 kDa); *lane 2*, human TMPRSS2 R255Q and human TMPRSS2 S441A mutant; *lane 3*, human wildtype TMPRSS2 and human TMPRSS2 S441A; *lane 4*, human wildtype TMPRSS2, human TMPRSS2 S441A, and human HAI-2. (**B**) The proteolytic activity of the Syrian hamster enzymes was checked by using a similar protocol as in the case of the human TMPRSS2 enzymes. The wildtype enzyme cleaved the inactive S440A mutant, while the R254Q mutant did not show any proteolytic activity. *Lane 1*, Kaleidoscope markers; *lane 2*, hamster TMPRSS2 R254Q and hamster TMPRSS2 S440A mutant; *lane 3*, hamster wildtype TMPRSS2 and hamster TMPRSS2 S440A; *lane 4*, hamster wildtype TMPRSS2, hamster TMPRSS2 S440A, and human HAI-2. (**C**) The proteolytic activity of the wildtype and zymogen R255Q mutant human TMPRSS2 was checked on the inactive one-chain hamster TMPRSS2 S440A protein substrate. The reaction mixtures were incubated for 0, 3, and 6 hours at 37°C. The RQ mutant enzyme cleaved the SA mutant protein at a 2:1 molar ratio (10.7 µM RQ and 5.35 μM SA), and this cleavage was completely inhibited by (1 µM) HAI-2. *Lane 1*, Kaleidoscope markers (250, 150, 100, 75, 50, 37, 25, 20, 15, 10 kDa); *lane 2*, human TMPRSS2 R255Q and hamster TMPRSS2 S440A mutant at 0 hour; *lane 3*, human TMPRSS2 R255Q and hamster TMPRSS2 S440A mutant at 3 hours; *lane 4*, human TMPRSS2 R255Q and hamster TMPRSS2 S440A mutant at 6 hours; *lane 5*, human TMPRSS2 R255Q, hamster TMPRSS2 S440A mutant, and HAI-2 at 0 hour; *lane 6*, human TMPRSS2 R255Q, hamster TMPRSS2 S440A mutant, and HAI-2 at 3 hours; *lane 7*, human TMPRSS2 R255Q, hamster TMPRSS2 S440A mutant, and HAI-2 at 6 hours; *lane 8*, hamster TMPRSS2 S440A; *lane 9*, human TMPRSS2 R255Q; *lane 10*, human wildtype TMPRSS2 and hamster TMPRSS2 S440A at 1 hour. (**D**) The proteolytic activity of the hamster wildtype and zymogen R254Q mutant TMPRSS2 was checked on the inactive one-chain hamster TMPRSS2 S440A protein substrate. The reaction mixtures were incubated for 0, 3, and 6 hours at 37°C. The RQ mutant enzyme cleaved the S440A mutant protein at a 2:1 molar ratio (10.7 µM RQ and 5.35 μM SA), and this cleavage was completely inhibited by (1 µM) HAI-2. *Lane 1*, Kaleidoscope markers (250, 150, 100, 75, 50, 37, 25, 20, 15, 10 kDa); *lane 2*, hamster TMPRSS2 R254Q and hamster TMPRSS2 S440A mutant at 0 hour; *lane 3*, hamster TMPRSS2 R254Q and hamster TMPRSS2 S440A mutant at 3 hours; *lane 4*, hamster TMPRSS2 R254Q and hamster TMPRSS2 S440A mutant at 6 hours; *lane 5*, hamster TMPRSS2 R254Q, hamster TMPRSS2 S440A mutant, and HAI-2 at 0 hour; *lane 6*, hamster TMPRSS2 R254Q, hamster TMPRSS2 S440A mutant, and HAI-2 at 3 hours; *lane 7*, hamster TMPRSS2 R254Q, hamster TMPRSS2 S440A mutant, and HAI-2 at 6 hours; *lane 8*, hamster TMPRSS2 S440A; *lane 9*, hamster TMPRSS2 R254Q; *lane 10*, hamster wildtype TMPRSS2 and hamster TMPRSS2 S440A at 1 hour. Hu, human; Ham, hamster.

To test whether the zymogen trans(auto)-activation mechanism applies for TMPRSS2, we incubated the human/Syrian hamster Arg255/254Gln (RQ) mutants with the Ser441/440Ala (SA) mutants and followed the proteolysis on SDS-PAGE. Our results show that the zymogen form of TMPRSS2 has very weak proteolytic activity. When we incubated the RQ and SA mutants at a 1:1 molar ratio, we could not see any proteolytic cleavage after incubation at 37°C for 1 hour (Figure 4.A,B). However, when we incubated the RQ mutants at high concentrations (10.7 µM) in a 2:1 molar excess with the hamster SA mutant (5.35 µM), after 3 and 6 hours, we could clearly detect the cleavage of the SA mutant (Figure 4C and D). Both the human and the hamster zymogens showed proteolytic activity that could be inhibited by HAI-2. Although the proteolytic activity of the zymogen TMPRSS2 is very weak (about 10,000-fold lower than that of the active enzyme), it should be sufficient to initiate the self-amplifying auto-activation process in the case of the wildtype enzyme.

**Figure 5:**
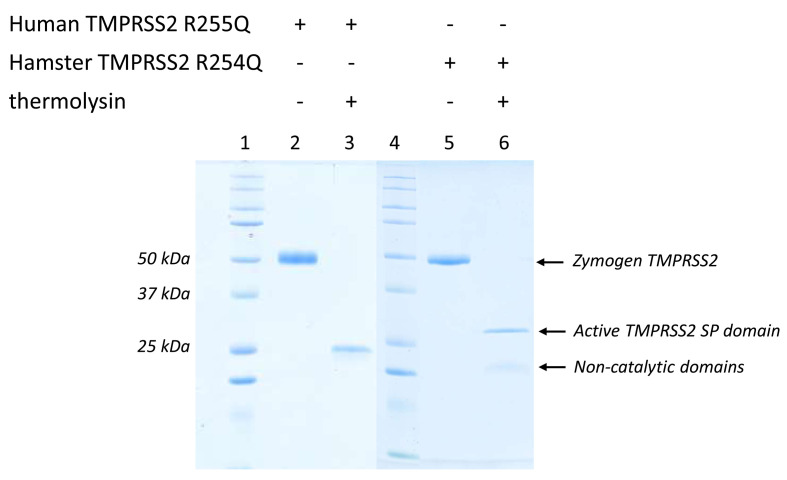
Thermolysin activates human TMPRSS2 R255Q and hamster TMPRSS2 R254Q zymogen mutants. The zymogen mutants were incubated with thermolysin at 0.1 m/m% for 5 hours at 37°C. The cleavage was detected on Coomassie-stained reducing SDS-PAGE. Thermolysin readily activated both human TMPRSS2 R255Q and hamster TMPRSS2 R254Q mutants. *Lane1 and Lane4,* Kaleidoscope (250, 150, 100, 75, 50, 37, 25, 20, 15, 10 kDa).

**Figure 6:**
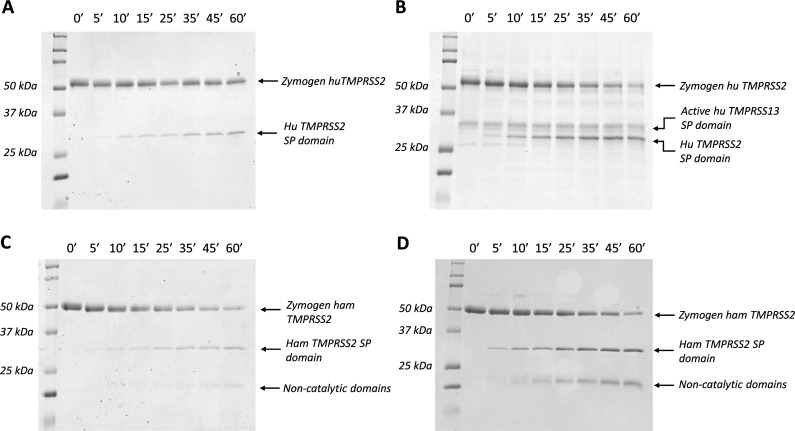
Time course of the cleavage of inactive TMPRSS2 S440/441A mutants by active TMPRSS-2/-13 enzymes. In order to determine the kinetic parameters of the autoactivation and the cross-activation reactions, we incubated the inactive one-chain S441A human (Hu) and S440A Syrian hamster (Ham) mutants with active proteases. Samples were taken at various time points (0, 5, 10, 15, 25, 35, 45, and 60 minutes) and appropriate amounts (about 1 µg human and 1.8 µg hamster protein/lane) were analyzed by SDS-PAGE under reducing conditions. (**A**) Cleavage of human TMPRSS2 S441A mutant by wildtype human TMPRSS2 enzyme. (**B**) Cleavage of human TMPRSS2 S441A mutant by wildtype human TMPRSS13 enzyme; (**C**) Cleavage of hamster TMPRSS2 S440A mutant by wildtype hamster TMPRSS2 enzyme; (**D**) Cleavage of hamster TMPRSS2 S441A mutant by thermolysin-activated hamster TMPRSS2 enzyme. *Lane 1,* Kaleidoscope (250, 150, 100, 75, 50, 37, 25, 20, 15, 10 kDa). Hu, human; Ham, hamster.

In order to make sure that our RQ mutants are correctly folded, we used thermolysin to proteolytically process the RQ mutants. Thermolysin is a bacterial metalloprotease that specifically cleaves at the N-terminal side of hydrophobic amino acid residues. Previously, we showed that limited proteolysis with thermolysin can specifically hydrolyze the Gln–Ile bond at the exposed activation site of the RQ mutants of trypsin-like proteases (e.g. C1r, MASP-2) [30,34]. We managed to selectively process the RQ mutant forms of both human and hamster TMPRSS2, which converted both into fully active SPs. In this way, we confirmed that these zymogen mutants are indeed correctly folded. The thermolysin-cleaved zymogen RQ mutants were active on both synthetic and protein substrates (Figures 5 and Figures 6.D, Supplementary Figure S2).

Next, we checked the second step, where the proteolytically processed, active enzyme molecules cleave the zymogens. We incubated the Ser441/440Ala zymogen mutants with already activated wildtype enzymes (Figure 4.A,B) and determined the kinetic parameters through densitometry of the SDS-PAGE gels (Figure 6.A,C, Table 2). Both the human and the hamster protease cleaved the Arg–Ile bond in the corresponding one-chain form very efficiently. The human TMPRSS2 proved to be more efficient in this reaction than the hamster one, but the data clearly demonstrate that both enzymes are capable of auto-activation. We also verified that HAI-2 is able to inhibit the auto-activation of TMPRSS2 (Figure 4.A,B).

**Table 2:** Cleavage rates of human (hu) TMPRSS2 /TMPRSS13, Syrian hamster (ham) TMPRSS2 on human TMPRSS2 S441A protein substrate and on Syrian hamster TMPRSS2 S440A protein substrate.

Enzyme	Hu TMPRSS2 S441A *k*_cat_/*K_M_* (M^–1^ s^–1^)	Ham TMPRSS2 S440A *k*_cat_/*K_M_* (M^–1^ s^–1^)
Wildtype, hu TMPRSS2	(1.77 ± 0.05) × 10^5^	-
Wildtype, ham TMPRSS2	-	(3.88 ± 0.70) × 10^4^
Thermolysin-activated ham TMPRSS2 R254Q	-	(1.46 ± 0.23) × 10^4^
Wildtype hu TMPRSS13	(2.48 ± 0.03) × 10^2^	(2.57 ± 0.58) × 10^1^

Kinetic assays were performed independently in triplicate, and standard deviations for each dataset are indicated

We also incubated the Ser441Ala mutants of human and hamster TMPRSS2 with a related TTSP, human TMPRSS13, to test the cross-activation mechanism. The proteolytically processed active TMPRSS13 protease readily cleaved zymogen TMPRSS2, although with less efficiency compared with the auto-activation reaction (Figure 6.B, Table 2). This experiment clearly demonstrates that a different TTSP can have similar enough substrate specificity to activate the zymogen of a different TTSP enzyme, supporting the proposed existence of a TTSP-based proteolytic network on the cell surface.

## Discussion

TMPRSS2 and other TTSPs as well are important drug targets in viral infections and various cancers. The drug development process was hindered due to the lack of adequate amounts of correctly folded, biologically active recombinant TMPRSS2.

Recently, several methods have been published for producing recombinant TMPRSS2; however, none of them yielded wildtype, biologically active ectodomain. Bacterial expression of the SP domain, either with renaturation or periplasmic expression, provided only a little amount of protein with low catalytic activity [35,36]. Eukaryotic expression procedures usually worked well for proteolytically inactive mutants; however, these were not suitable for producing the wildtype protease [6,23]. Fraser et al. expressed recombinant wildtype TMPRSS2 ectodomain in a baculovirus-insect cell system [27]. In their construct (dasTMPRSS2), however, they replaced the SRQSR255 activation peptide with the enterokinase cleavage sequence DDDDK255. While their recombinant protein was produced in zymogenic form and could be activated by enterokinase, it is definitely not entirely wildtype. McCallum et al. expressed a recombinant ectodomain construct in Expi293 human cells [37]. Their starting point was the baculovirus construct (dasTMPRSS2), but they introduced several mutations (e.g. the T447C and the restoration of the N249 glycosylation site by inserting two extra serine residues) and added an enterokinase-cleavable N-terminal SUMO tag to the sequence. With these modifications, they managed to get active, mutant TMPRSS2 ectodomain in Expi293 cells by means of transient transfection.

Unlike the above approaches, our construct does not contain any mutations in the coding sequence of the TMPRSS2 ectodomain; we managed to elaborate a method to produce fully active wildtype TMPRSS2 ectodomain in mammalian cells (HEK, CHO). The novelty of our method is that we prevented the activation of the TMPRSS2 ectodomain during the expression and secretion process by co-expressing it with the cognate inhibitor HAI-2, which, and this is another important feature, remains withheld in the plasma membrane. The results we obtained unequivocally demonstrate that our recombinant TMPRSS2 ectodomain is perfectly folded, as it has an extremely high catalytic activity, which is comparable to that of trypsin. Such a high catalytic activity, if uncontrolled, can degrade the recombinant protein in the secretory pathway and can also be toxic to the host cell. The high degree of autolysis we demonstrated in this study and cellular toxicity together could be the reason for the failure of previous attempts to produce recombinant wildtype TMPRSS2.

HAI-1 and HAI-2 are type I transmembrane proteins having a C-terminal intracellular part and an N-terminal extracellular region with two Kunitz-type inhibitor domains. Although it has not been fully characterized yet, apparently, these molecules play an important role in the regulation of the TTSP network on cell surfaces. The fact that HAI-1 or HAI-2 deficiency both cause embryonic lethality in mouse models highlights their physiologic significance [38–40]. Moreover, in the case of two related TTSPs, matriptase and TMPRSS13, it has been shown that HAI-1 and HAI-2 may act as chaperones in the secretory pathway, helping the correct folding and membrane positioning of these two proteins [25,41,42].

Since it was shown that HAI-2 inhibits TMPRSS2 more efficiently than HAI-1 [8], we chose HAI-2 as a co-expression partner for the recombinant protease production. This expression strategy perfectly solved all problems we previously encountered for both the human and Syrian hamster TMPRSS2, and also for the related TTSP, TMPRSS13. We note that for the expression of the highly autocatalytic recombinant complement SP MASP-1, Degn et al. used a similar strategy [43]. They produced full-length recombinant human zymogen MASP-1 in a mammalian expression system through co-expressing it with its natural negative regulator, the C1-inhibitor. We suggest the general conclusion that highly active, autocatalytic proteases can be successfully produced in recombinant form when co-expressed with their physiological inhibitors.

We note that our results are not in line with a chaperone role for HAI-2. The inactive zymogen TMPRSS2 ectodomain mutants were efficiently expressed and secreted by the HEK293F cells in the absence of HAI-2. Co-expression with HAI-2 did not increase the amount of the recombinant protein in this case; on the contrary, it reduced the yield, probably because the cells had to produce two different recombinant proteins at the same time. In the case of the wildtype TMPRSS2, however, the presence of HAI-2 during the expression and secretion turned out to be an absolute necessity. We can draw the conclusion that the only role of HAI-2 is to prevent the activation of the protease during secretion (e.g. in the ER and in the Golgi), i.e. it does not influence the folding of the nascent protein or the transport of the protein to the cell surface.

Another important observation is that the secreted wildtype TMPRSS2 ectodomain is present predominantly in the one-chain zymogen form in the medium immediately after secretion (Figure 2.A). The zymogen auto-activates during purification and concentration (Figure 2.B,C). After purification, we can detect the intact SP domain and the degradation products of the LDLR-A and SRCR domains on reduced SDS-PAGE. Our results agree with those of Fraser et al., who had detected the same pattern for their recombinant TMPRSS2 ectodomain (dasTMPRSS2) expressed in Sf9 insect cells [27].

Our results, however, do not support the idea that the majority of TMPRSS2 activates inside the mammalian cells before reaching the cell surface. If we express the protease without the inhibitor, only trace amounts of recombinant protein can be detected in the supernatant; the majority of the TMPRSS2 gets stuck and degraded inside the cells. If we express the protease together with the inhibitor, it leaves the cell, but predominantly in zymogen form. Our findings suggest that under physiological conditions, the activation of zymogen TMPRSS2 should occur on the cell surface either through zymogen trans(auto)-activation or through cross-activation by other TTSPs. These findings also support the notion that natural TMPRSS2-producing cells also co-express suitable inhibitors (i.e. HAI-1 and HAI-2) that suppress intracellular activation [44].

A striking difference between the human and the hamster TMPRSS2 ectodomain is the presence of an extra N-glycosylation site (N249) in the human sequence. As a consequence, the human TMPRSS2 ectodomain is glycosylated at two sites: N213 in the SRCR domain and N249 in the activation peptide of the SP domain, while the hamster TMPRSS2 molecule is glycosylated only in the SRCR domain. To test the role of the N249 glycosylation of the human protein, we made a mutation that abolished the N249 glycosylation (S251A). This mutation completely prevented the secretion of the human recombinant TMPRSS2 ectodomain (data not shown). The fact that the N249 glycosylation is absolutely necessary for the recombinant expression of the human TMPRSS2 ectodomain is quite unexpected, especially when we consider that this post-translational modification is absent from the highly similar hamster protein, and yet, it is secreted very efficiently by the HEK293F cells.

Our results are in disagreement with those of Zhang et al., who found that abolishing N-glycosylation at N213, but not N249, prevents full-length human TMPRSS2 from exiting the ER of the HEK293 cells [17]. On the other hand, McCallum et al. also demonstrated that the presence of the N249 glycosylation is a prerequisite for efficient recombinant human TMPRSS2 ectodomain expression in mammalian cells [37].

During zymogen activation of trypsin-like SPs, an (Arg/Lys)–(Ile/Val) bond is cleaved in the SP domain, followed by a conformational change that forms the active site with the oxyanion hole and the substrate binding pocket [45]. This conformational change can take place in the one-chain zymogen form as well, although only with low frequency and only for a short time [46]. While the zymogen form oscillates between the inactive and the active conformations, the equilibrium is usually strongly shifted to the inactive state [47]. In the case of proteases capable of zymogen trans(auto)-activation, the proportion of zymogen molecules getting into the active conformation is usually high enough to result in measurable proteolytic activity [32]. The zymogenicity factor is a numerical value that can be used to characterize the potential of proteases to auto-activate. It is the ratio of the catalytic activities of the activated versus the zymogen forms on the zymogen substrate. The zymogenicity among the proteases can vary within wide limits: it is very high for trypsin (~10^7^) [48], and it could be very low for some auto-activating proteases such as matriptase (in the range of 27-33) [27–33,49] and tissue-type plasminogen activator (in the range of 5-10) [5–10,50].

However, one should be aware that zymogenicity alone is not enough to judge the auto-activation properties *in vivo*. For example, while trypsins have high zymogenicity, these enzymes can auto-activate. As activated trypsin is a highly potent trypsinogen activator, even trace amounts of active trypsin can be dangerous in the pancreas, as intrapancreatic auto-activation of trypsinogen can cause pancreatitis. As a natural defense mechanism, the acinar cells of the pancreas secrete SPINK1 (serine protease inhibitor Kazal type 1), which protects the pancreas from ectopic auto-activation of trypsinogen [51]. Trypsinogen activation, therefore, normally starts outside of the pancreas, in the lumen of the duodenum, where enterokinase cross-activates trypsinogen to trypsin, initiating a self-amplifying auto-activation process.

In these respects, TMPRSS2 appears to be more similar to trypsin than to matriptase: just like trypsinogen, the TMPRSS2 zymogen also has only very low protease activity. We estimate that its activity is 10,000-fold weaker than that of the active enzyme. In other words, the zymogenicity factor of TMPRSS2 is in the 10^4^ range. To detect the proteolytic activity of the zymogens, we had to incubate the RQ mutants with the SA mutant substrate at high concentration (10.7 µM) in 2:1 molar excess for 3–6 hours at 37°C (Figure 4.C,D). The proteolytic activity of the stable zymogen RQ mutants was very weak in our experimental system; however, *in vivo,* the local concentration of the TMPRSS2 zymogen (e.g. in the secretory pathway and on the cell surface) should be high enough to initiate the auto-activation process. On the other hand, both the human and the hamster active wildtype TMPRSS2 cleave the SA mutants with high efficiency (*k*_cat_/*K*_*M*_ is in the 10^4^–10^5^ M^-1^s^-1^ range), which indicates that the zymogen form of the enzyme is a very good substrate for the active protease. It means that, similarly to trypsin, even traces of active TMPRSS2 can convert large amounts of zymogen TMPRSS2 into the active form quickly and efficiently. This similarity to trypsin might extend to the role of TMPRSS2 inhibitors that prevent premature zymogen auto-activation and potentially also to the existence of cross-activator proteases at the location where TMPRSS2 needs to function. In this study, we already demonstrated that another cognate protease (TMPRSS13) can activate the zymogen TMPRSS2. Both TMPRSS2 and TMPRSS13 are robustly expressed in cells of the human upper airway and can promote SARS-CoV-2 infection. It is reasonable to assume that these closely related proteases can cross-activate each other’s zymogen forms *in vivo*.

The activation mechanism of the TTSPs is a controversial topic in the literature. For example, Skovbjerg et al. demonstrated that the zymogen one-chain matriptase has significant enzymatic activity, which can be inhibited by HAI-1 and HAI-2 [52]. They proposed that zymogen activation occurs by trans(auto)-activation between two matriptase zymogen molecules. This is the mechanism, which we proved in the case of TMPRSS2 as well, although the enzymatic activity of the one-chain zymogen TMPRSS2 mutant is much lower than that of the corresponding matriptase mutant (R614A). Another publication claims that zymogen matriptase is activated by TMPRSS2 [6]. These authors showed that during prostate cancer progression, TMPRSS2 plays a role in matriptase activation. They propose that TMPRSS2 initiates a pericellular proteolytic cascade to activate matriptase.

**Figure 7:**
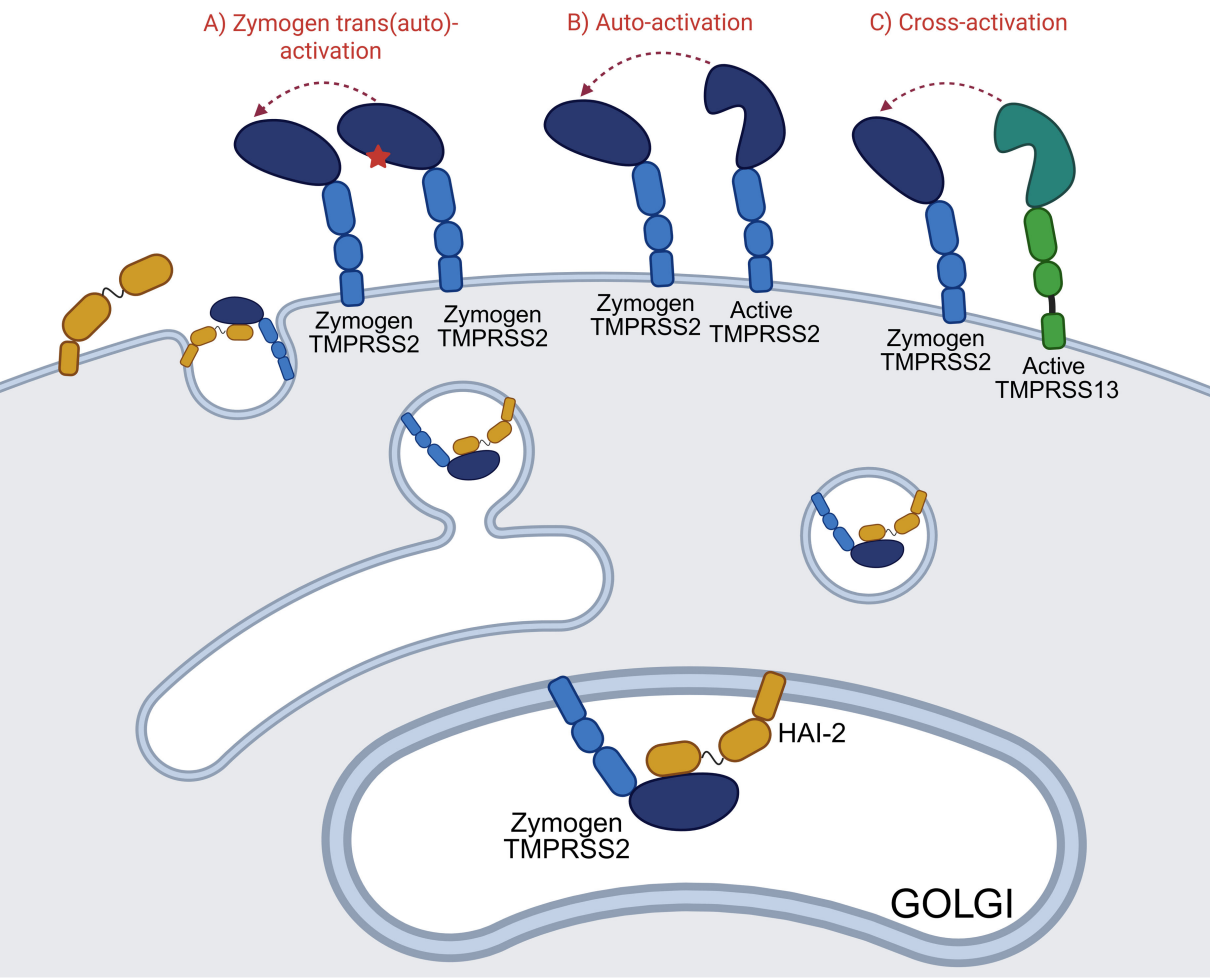
Schematic representation of the activation mechanism of zymogen TMPRSS2 at the cell surface. The HAI-2 inhibitor prevents premature activation of zymogen TMPRSS2 in the ER and Golgi during its maturation and trafficking to the cell surface by forming a transient complex with the zymogen protease. We also exploit this phenomenon during recombinant ectodomain expression. Once at the membrane, there are three possible activation mechanisms: (**A**) Zymogen trans(auto)-activation, in which a one-chain zymogen TMPRSS2 in an active-like conformation (red asterisk) cleaves another zymogen molecule; (**B**) Auto-activation, in which an active, two-chain TMPRSS2 cleaves a zymogen TMPRSS2 nearby; or (**C**) Cross-activation, in which another TTSP, active TMPRSS13, cleaves zymogen TMPRSS2.

We propose that the two activation mechanisms are not mutually exclusive. In the case of proteases where the zymogen has significant proteolytic activity, the zymogen trans(auto)-activation mechanism dominates, while in the case of proteases with barely detectable zymogen activity, the initiation step could be executed through cross-activation by a different protease. Our results show that the ectodomain of TMPRSS2, if co-expressed with HAI-2, is secreted predominantly in the one-chain zymogen form. It seems plausible to assume that *in vivo,* a significant part of the nascent TMPRSS2 is activated on the cell surface (Figure 7).

Previously, we studied the auto-activation mechanism of the complement lectin pathway proteases, demonstrating that the one-chain zymogens (RQ mutants) of both MASP-1 and MASP-2 have measurable enzymatic activity and proved the zymogen trans(auto)-activation mechanism [32]. MASP-1 has significant zymogen activity, while zymogen MASP-2 proteolytic activity is barely detectable. *In vitro,* both proteases are capable of auto-activation, but under physiological conditions in the normal human blood, MASP-1 is the exclusive activator of zymogen MASP-2 [53]. Similar mechanisms might also exist in the case of the cell-surface proteolytic cascades as well.

In conclusion, we developed a method by which native, enzymatically active recombinant TMPRSS2 ectodomain can be produced in mammalian expression systems. This method is most likely suitable for the recombinant expression of other TTSPs that activate through a zymogen trans(auto)-activation mechanism, as we already demonstrated through the expression of TMPRSS13.

We determined the kinetic parameters for the hydrolysis of synthetic and protein substrates and found that the enzymatic activity of the recombinant TMPRSS2 ectodomain is very high; it is comparable to that of trypsin. HAI-2, a cognate inhibitor of the TTSPs, inhibited the enzymatic activity of TMPRSS2 of both species with picomolar *K*_*I*_ values, i.e. HAI-2 is a tight-binding inhibitor of both human and hamster TMPRSS2. In some instances, tight-binding interactions coincide with a slow-binding mechanism where the inhibitor, the enzyme, or both need to go through a slow conformational change prior to (conformation-selection mechanism) or during (induced fit mechanism) complex formation. As in this study, we focused on the production of fully active TMPRSS2 and on deciphering its activation mechanism. We did not test whether there is a slow-binding interaction at play; nevertheless, we applied a very long incubation time to achieve binding equilibrium for the interaction.

We also showed that active TMPRSS2 cleaves its zymogen form with high efficiency, proving that it is an auto-activating protease. The zymogen form of TMPRSS2 has a low, but clearly detectable proteolytic activity, which is apparently sufficient for initiating a zymogen auto-activation process. The fact that TMPRSS13 can cross-activate zymogen TMPRSS2 supports the idea that the TTSPs form a proteolytic network on the cell surface (Figure 7). Our procedure offers a new, general way to produce fully active wildtype TTSP enzymes and can boost the development of highly potent and selective TMPRSS2 and other TTSP inhibitors.

## Materials and methods

### Designing and cloning the recombinant constructs

Synthetic genes encoding the soluble ectodomains of human TMPRSS2 (UniProt identifier O15393-1, residues 107–492) and hamster TMPRSS2 (UniProt identifier A0A1U8C7 × 1, residues 108–491) were synthesized (GeneArt, Thermo Fisher Scientific) and subcloned into the pSecTag2 expression vector (Invitrogen) using standard molecular biology techniques. Each construct contained an N-terminal Ig κ-chain signal peptide to ensure secretion and C-terminal Myc and hexahistidine (His₆) tags. The human TMPRSS2 also displayed an additional FLAG epitope tag at the N-terminus.

Site-directed mutagenesis was performed using a whole plasmid amplification strategy to generate specific point mutations in TMPRSS2: R255Q, S441A, and S251A for the human protein and R254Q and S440A for the hamster variant. The mutagenesis reactions were performed using PCRBIO HiFi polymerase enzyme (PCR Biosystems, U.K) and the following primers: for human TMPRSS2 R255Q: 5′AGCAGCCGTCAGAGCCAGATTGTTGGTGGCGAAAGC3′, and 5′GCTTTCGCCACCAACAATCTGGCTCTGACGGCTGCT3′; for human TMPRSS2 S441A: 5′AGCTGTCAGGGTGATGCTGGTGGTCCGCTGGTT3′ and 5′AACCAGCGGACCACCAGCATCACCCTGACAGCT3′; for human TMPRSS2 S251A: 5′GTCAACCTGAACTCCGCCAGGCAGTCTCAAATCG3′ and 5′CGATTTGAGACTGCCTGGCGGAGTTCAGGTTGA C3′; for hamster TMPRSS2 R254Q: 5′CGACACGCCAGAGCCAGATTGTGGGTGGATCGA3′ and 5′TCGATCCACCCACAATCTGGCTCTGGCGTGTCG3′; for hamster TMPRSS2 S440A: 5′TTGCCAGGGAGACGCTGGAGGGCCTTTGGTTAC3′ and 5′GTAACCAAAGGCCCTCCAGCGTCTCCCTGGCAA3′. The resulting products were then digested with the DpnI enzyme (Thermo Fisher Scientific) and transformed into TOP10 competent cells.

The synthetic gene encoding the human TMPRSS13 ectodomain (UniProt Identifier Q9BYE2-1, residues 187–586) was cloned similarly to the TMPRSS2 into the pSecTag2 vector, featuring an N-terminal Ig κ-chain signal peptide and a C-terminal His₆ tag.

Human HAI-2 constructs (UniProt Identifier O43291-1) (full-length and ectodomain) were synthesized (GeneArt, Thermo Fisher Scientific) and expressed using the pcDNA3.1 vector. The HAI-2 ectodomain construct encoded the residues 36–196 and included a C-terminal His₆ tag.

The SARS-CoV-2 S protein ectodomain construct (UniProt Identifier P0DTC2) was recombinantly expressed in insect cells as described in Fernandes et al. [26] with minor modifications. Briefly: a modified spike construct was designed containing stabilizing mutations K968P and V987P, a truncation after residue P1213, and insertion of the HIV gp160-derived foldon trimerization domain after Q1033. This was followed by a C-terminal His₆ tag. The multibasic S1/S2 cleavage site was preserved. The gene was synthesized by Gene Art (Thermo Fisher Scientific) and subcloned into the baculovirus transfer vector pOET3 under the control of the AcMNPV basic (p6.9) promoter. Recombinant baculoviruses (rBac) were generated using the flashBAC GOLD™ system (Oxford Expression Technologies, U.K), following the manufacturer’s protocol.

### Cell culture and transfection

Recombinant TMPRSS2 (human and hamster), TMPRSS13, and HAI-2 proteins were expressed in FreeStyle™ 293 F cells (Thermo Fisher Scientific), a suspension-adapted derivative of the HEK293 cell line. Cells were maintained at 37°C in 8% CO₂ in FreeStyle™ 293 Expression Medium and co-transfected using 293fectin™ (Thermo Fisher Scientific) according to the manufacturer’s instructions. Transfections were performed in serum-free medium, and cultures were incubated for 96–120 hours post-transfection before harvest.

S protein production was carried out in insect SuperSf9 cells grown in suspension in 300 ml shake flasks. Cells were seeded at a density of 1.2 × 10⁶ cells/ml and infected with rBac at a cell density corresponding to 2 × 10⁶ cells/ml. Cultures were incubated at 27°C and monitored daily for viability and cell density. Harvest was performed 72 hours post-infection when viability dropped below 70%.

### Protein purification

Conditioned media from both mammalian and insect expression systems were collected by centrifugation at 200 g for 10 minutes. The supernatant was filtered through a 0.45 µm membrane (Sarstedt), followed by buffer exchange using G25 preparative gel filtration chromatography (Cytiva).

The filtered supernatants were subjected to IMAC via Ni-NTA agarose resin (Qiagen) equilibrated with binding buffer (20 mM HEPES, 300 mM NaCl, 20 mM imidazole, pH 7.4). Bound proteins were eluted using binding buffer containing 250 mM imidazole. Further purification was carried out by size-exclusion chromatography on a preparative Superose 12 Prep Grad column (Cytiva) equilibrated with 20 mM HEPES, 145 mM NaCl, pH 7.4. Eluted fractions were analyzed, pooled, and concentrated using a centrifugal filter device (10 kDa MWCO; Millipore). For the determination of the concentration of the purified proteins, we used the following extinction coefficients at 280 nm: human TMPRSS2 ectodomain 2.348 ml mg^-1^ cm^-1^; hamster TMPRSS2 ectodomain 2.349 ml mg^-1^ cm^-1^.

### Western blot analysis

Aliquots of culture supernatants and cell pellets were collected and analyzed by SDS-PAGE followed by western blotting. Proteins were transferred to nitrocellulose (Bio-Rad) membranes and probed using a primary anti-human TMPRSS2 monoclonal antibody (MA550445, Thermo Scientific) at a dilution of 1:1500. HRP-conjugated anti-rabbit IgG secondary antibody (31460, Invitrogen) was used at a dilution of 1:10,000. Signal detection was performed using an enhanced chemiluminescence (Clarity Western ECL Substrate) system (Bio-Rad).

### Enzymatic measurements

#### Enzymatic activity on synthetic substrate

The activities of recombinant TMPRSS2 ectodomain constructs were tested in black non-binding microtiter plates (655900, Greiner Bio-One) using 50 µM Boc-Gln-Ala-Arg-AMC (4017019, Bachem) as a fluorogenic substrate. Cleavage reactions were performed in assay buffer (20 mM HEPES, pH 7.4, 145 mM NaCl, 0.05% Triton X-100) at 30°C, with fluorescence measured at excitation/emission wavelengths of 380/460 nm, respectively.

The *k*_cat_ and *K*_*M*_ values of the active enzymes were determined using the same fluorogenic assay. Initial velocities (*v₀*) were measured with 4 nM TMPRSS2 ectodomain and serial dilutions of the Boc-Gln-Ala-Arg-AMC substrate. Michaelis–Menten curves were generated, and kinetic parameters were derived by nonlinear regression fitting using Origin software.

#### Active site titration and equilibrium inhibitory constant determination

Our preliminary tests on the interaction of HAI-2 with both the human and the hamster TMPRSS2 indicated an *IC_50_* value of HAI-2 close to the concentration of the enzymes, which is a characteristic of a tight-binding inhibition scenario. Therefore, instead of a Michaelis–Menten kinetic model, which assumes a large molar excess of the inhibitor at 50% enzyme inhibition, the Morrison approach had to be applied [54]. As Kuzmic et al. demonstrated, both the active enzyme concentrations and the equilibrium inhibitory constant can be determined by non-linear fitting to the Morrison equation below:


$$V_{I}\text{=}\left(V_{0}\text{/}2E_{T}\right)\left(\left(E_{T}-I_{T}-K_{I}\right)+\sqrt{{\left(E_{T}-I_{T}-K_{I}\right)}^{2}+4E_{T}K_{I}}\right)$$

if certain conditions are provided [55]. In the equation *V*_0_ and *V_I_* are the rates of the enzymatic reaction in the absence or in the presence of inhibitor, respectively (after subtracting the rate of spontaneous hydrolyzation of the substrate), *E_T_* and *I_T_* are the total active enzyme and total inhibitor concentrations, respectively, and *K_I_* is the equilibrium inhibitory constant.

As our laboratory routinely uses the P1 M84R variant of *E. coli* ecotin for active site titration of trypsin-like proteases, at first step, we used that inhibitor to determine the concentration of active human and hamster TMPRSS2 [56]. A two-fold serial dilution of the inhibitor (ranging from 40 nM to 156 pM) was incubated with 10 nM of human or hamster TMPRSS2 enzyme for 2 hours at room temperature. *V*_0_ and *V_I_* values were then measured using 250 µM Boc-Gln-Ala-Arg-AMC and plotted against inhibitor concentrations. Nonlinear curve fitting was performed in Origin software using the above equation, where *E_T_* and *K_I_* were the two adjustable parameters. The experimentally determined enzyme concentration (*E*_*T*_) was then compared with the theoretical concentration calculated from absorbance at 280 nm.

To determine the inhibitory constant for HAI-2, 50 pM human or hamster TMPRSS2 was incubated with a 2/3-fold serial dilution of the inhibitor, ranging from 300 to 5.2 pM. Incubation time was extended to 16 hours at room temperature. *V*_0_ and *V_I_* values were measured, and *K*_*I*_ values were determined by curve fitting as described above.

#### TMPRSS2 proteolytic activity on S protein

Recombinant ectodomains of human and hamster TMPRSS2 (at final concentrations of 33  nM and 100  nM, respectively) were incubated with the intact ectodomain of the S protein (10  µM). Reactions were carried out under physiological pH and ionic strength buffer conditions (20 mM HEPES, 145 mM NaCl, pH 7.4) at 37°C for 1 hour. Proteolytic processing was assessed by SDS-PAGE. As a negative control, TMPRSS2 was preincubated with 1 µM HAI-2 before adding to S protein to prevent proteolytic cleavage.

#### Testing the proteolytic activity of the R255Q/R254Q zymogen mutants

The proteolytic activity of the one-chain RQ zymogen mutants was tested on the one-chain SA mutant substrates. Human and hamster proteolytically processable but catalytically inactive TMPRSS2 S441A/S440A (SA) mutants were incubated with the stable zymogen R255Q/R254Q (RQ) mutants at a 1:1 molar ratio (human: 0.75  μM, hamster: 10  μM) in 20 mM HEPES, 145 mM NaCl, pH 7.4 buffer for 60 minutes at 37°C. Following incubation, the samples were analyzed by SDS-PAGE to determine whether proteolytic cleavage of the TMPRSS2 S441A/S440A mutants had occurred, as indicated by the appearance of the SP domain fragment. As controls, the SA mutant substrates were also incubated with wildtype enzymes for 60 minutes at 37°C in the presence and absence of HAI-2. The concentrations used in these control reactions matched those described in the kinetic measurements. The experiment was also performed at higher protein concentrations and for longer incubation times. The catalytically inactive hamster TMPRSS2 S440A mutant was incubated with the stable zymogen R255Q/R254Q mutants at a 1:2 molar ratio (5.35 μM SA and 10.7 µM RQ) in 20 mM HEPES, 145 mM NaCl, pH 7.4 buffer for 180 and 360 minutes at 37°C, both in the presence and absence of 1 µM HAI-2.

#### Determining the kinetic constants of autocatalytic activation

Auto-activation assays were performed under physiological pH and ionic strength buffer conditions (20 mM HEPES, 145 mM NaCl, pH 7.4). Human and hamster TMPRSS2 S441A/S440A inactive zymogen mutants serving as substrates were prepared at concentrations of 1.97 μM and 3.36 μM, respectively. Wildtype active human and hamster (33 nM/100 nM) TMPRSS2 ectodomains were incubated with the SA zymogen mutants, and aliquots (*n*=7) were withdrawn over a 60-minute time course to monitor proteolytic conversion of the one-chain molecules. The consumption of the intact TMPRSS2 and the release of the SP domain fragment were detected on reducing SDS-PAGE. Kinetic constants were determined by densitometric quantification of the intact TMPRSS2 using a UVITEC Alliance imaging system and Image Lab software (BioRad). Experiments were conducted in triplicate for both wildtype enzymes. The reactions were assumed to be of the Michaelis–Menten type. The decay of the intact TMPRSS2 SA mutant was plotted over time, and the catalytic efficiency (*k*_cat_/*K*_*M*_) was estimated by nonlinear regression in Origin software using the following equation:


$$Y=A\times e\frac{x}{t}+Y_{0}$$

where *Y*_0_ represents the initial substrate concentration, and *A* and *t* are parameters obtained from the curve. The catalytic efficiency was then calculated as *k*_cat_/*K*_*M*_=(*E*_total_ × *t*)^-1^. In the presence of 0.67 µM human HAI-2, no cleavage could be detected, and therefore this reaction was used as a negative control.

#### Cross-activation of TMPRSS2 zymogen with TMPRSS13

In the cross-activation experiments, active human TMPRSS13 (1 µM) was used to cleave human (1.97  µM) and hamster (3.36 µM) TMPRSS2 SA mutant zymogens over a 120-minute incubation time. Aliquots were collected at eight time points throughout the reaction. Proteolytic activation was monitored by reducing SDS-PAGE by following the intact TMPRSS2. Kinetic parameters were calculated by densitometric analysis as described previously.

#### Testing the proteolytic activity of the thermolysin-activated TMPRSS2 R254Q mutant

The R255Q/R254Q (3.2 µM) mutants were activated by thermolysin as described in Gál et al. [30]. Activation assays were performed using thermolysin-activated hamster TMPRSS2 R254Q mutant. In these experiments, the substrate (hamster S440A mutant zymogen) concentration was set at 3.88 μM, while the active enzyme was present at a final concentration of 39 nM. The reaction proceeded for 60 minutes, during which seven aliquots were collected at specified time points.

Proteolytic activation was monitored by reducing SDS-PAGE, following the same methodology as previously described. Kinetic parameters were determined by densitometric analysis of the intact TMPRSS2, calculated according to the procedures outlined earlier.

## Supplementary material

online supplementary material 1.

## Data Availability

The sequences of the synthetic genes encoding the recombinant proteins were deposited in the Figshare database (https://portlandpress.figshare.com) with identifier number DOI: 10.6084/m9.figshare.30271975 [57]. All other data are included in the main manuscript text and the supplementary file.

## Open Access

This article has been published open access under our Subscribe to Open programme, made possible through the support of our subscribing institutions, learn more here: https://portlandpress.com/pages/open_access_options_and_prices#conditional

## Abbreviations

AcMNPV: Autographa californica Multiple Nucleopolyhedrovirus
CHO: Chinese Hamster Ovary
CMV: cytomegalovirus
HAI: hepatocyte growth factor activator inhibitor
HCoV-229E: human coronavirus 229E
HPR: Horseradish Peroxidase
IMAC: immobilized metal affinity chromatography
LDLR-A: low-density lipoprotein receptor domain class A
MASP: mannose-binding lectin-associated serine protease
MBP: maltose-binding protein
MERS-CoV: Middle East respiratory syndrome coronavirus
RQ mutant: Stable zymogen mutant in which the arginine of the scissile bond (R255 in human, R254 in hamster) is mutated to glutamine.
S: spike protein
SARS-CoV: severe acute respiratory syndrome coronavirus
SA mutant: Zymogen mutant in which the catalytic serine (S441 in human, S440 in hamster) is mutated to alanine.
SP: serine protease
SRCR: scavenger receptor cysteine-rich domain
TM: transmembrane domain
TMPRSS2: transmembrane protease serine 2
TTSP: type II transmembrane serine protease
rBac: recombinant baculovirus
