# Supplementary material for: Efficient production of fully active, SARS-CoV-2-priming, wildtype TMPRSS2 ectodomain via co-expression of HAI-2 allows for both auto- and cross-activation mechanisms
Source: Biochem J. 2025 Dec 17;482(24):1993–2010. doi: 10.1042/BCJ20253453 (PMC12794319; doi:10.1042/BCJ20253453)
Supplement: online supplementary material 1. [file bcj-482-24-BCJ20253453-s001.docx]

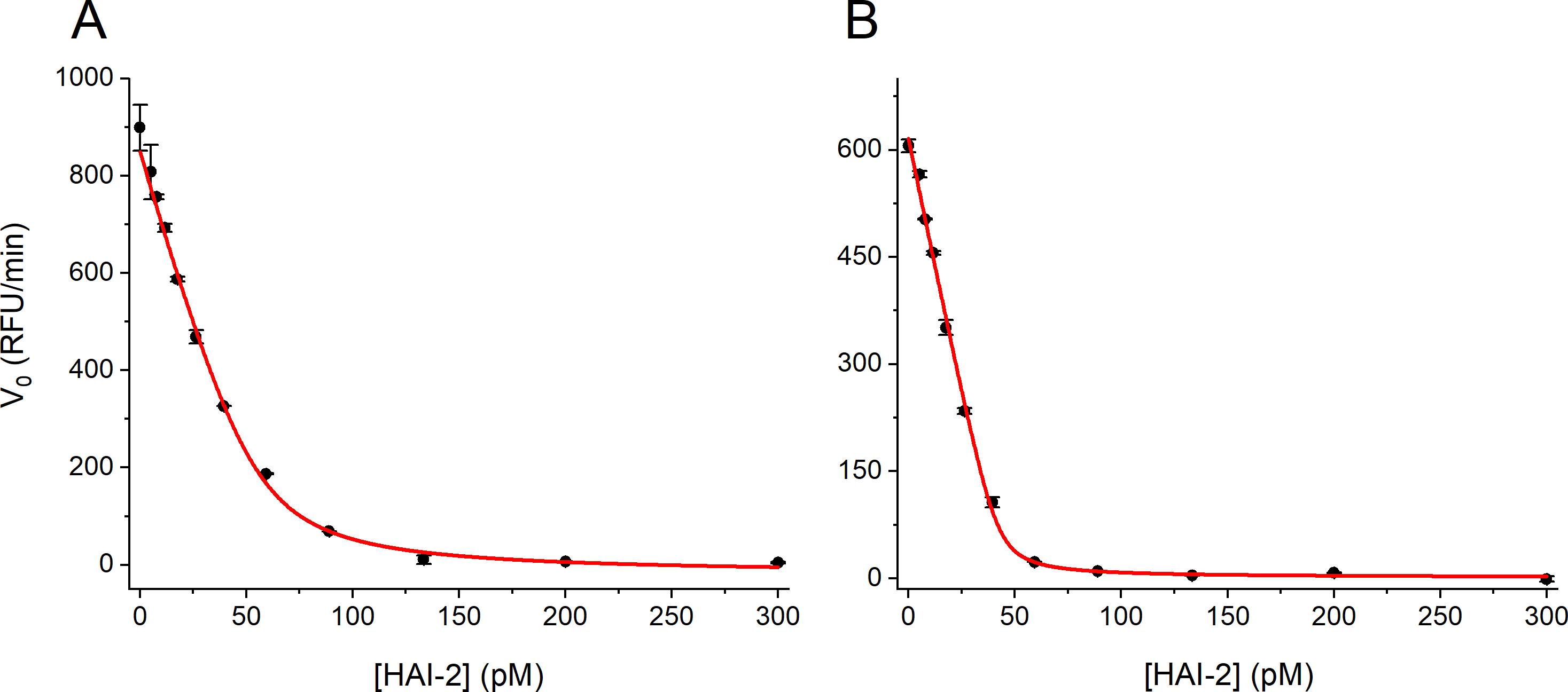


**Supplementary Fig. 1. Equilibrium inhibitory constant determination** 50 pM human **(A)** or hamster **(B)** TMPRSS2 was incubated for 16 hours at room temperature with a 2/3-fold serial dilution of HAI-2 ranging from 300 to 5.2 pM. Residual enzymatic activity was measured using 50 µM Boc-Gln-Ala-Arg AMC substrate. Data points represent mean ± SD while the red line shows the non-linear curve fit to the Morrison equation.


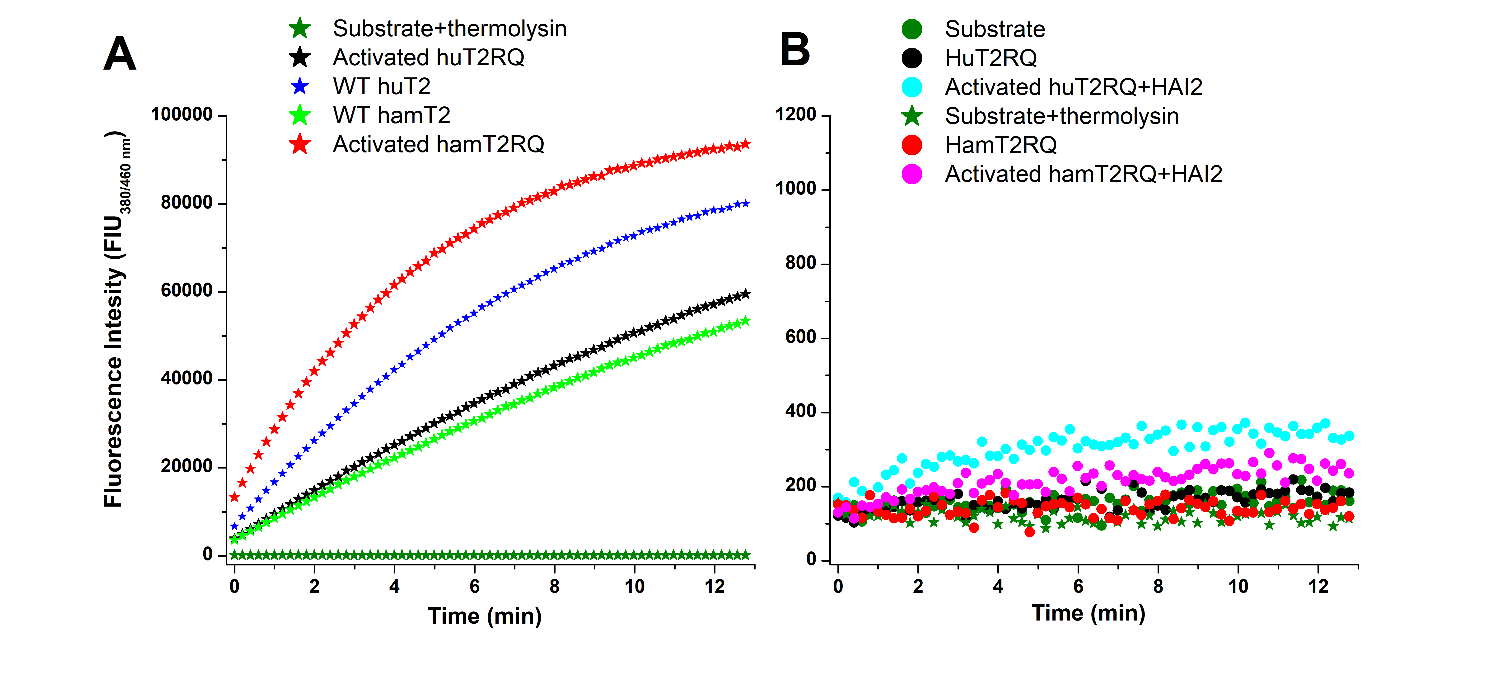


**Supplementary Fig. 2. Activity measurements of thermolysin-activated human and Syrian hamster TMPRSS2 R255(254)Q mutant ectodomains.** **(A)** The fluorogenic substrate Boc-Gln-Ala-Arg-AMC (50 µM) was incubated with 5 nM of the following proteins: thermolysin-activated human TMPRSS2 R255Q mutant (black asterisk), wild-type human TMPRSS2 (blue), thermolysin-activated hamster TMPRSS2 R254Q mutant (red), and wild-type hamster TMPRSS2 (light green). Generation of the AMC fluorophore was monitored over time. The RQ mutants exhibited activity levels comparable to their respective wild-type counterparts, with minor differences possibly due to variation between protein lots. **(B)** One-chain, non-activated human R255Q (black) and hamster R254Q (red dot) mutants (5 nM) showed no detectable substrate cleavage. Similarly, no activity was observed for the thermolysin-activated human (light blue) or hamster (magenta) RQ mutants in the presence of 160 nM HAI-2 inhibitor.

Background fluorescence is indicated by substrate incubated with thermolysin (dark green asterisk in panels A and B) or substrate alone (dark green dot in panel B). All measurements were performed in triplicate; a single representative dataset is shown in both panels.
